# Supplementary material for: Rapid Automated Screening for SARS-CoV-2 B.1.617 Lineage Variants (Delta/Kappa) through a Versatile Toolset of qPCR-Based SNP Detection
Source: Diagnostics (Basel). 2021 Oct 1;11(10):1818. doi: 10.3390/diagnostics11101818 (PMC8534837; doi:10.3390/diagnostics11101818)
Supplement: Supplementary file 1 [file diagnostics-11-01818-s001.zip › diagnostics-1376430-supplementary/210824_supp1_curves.pdf]

Supplementary figure S1

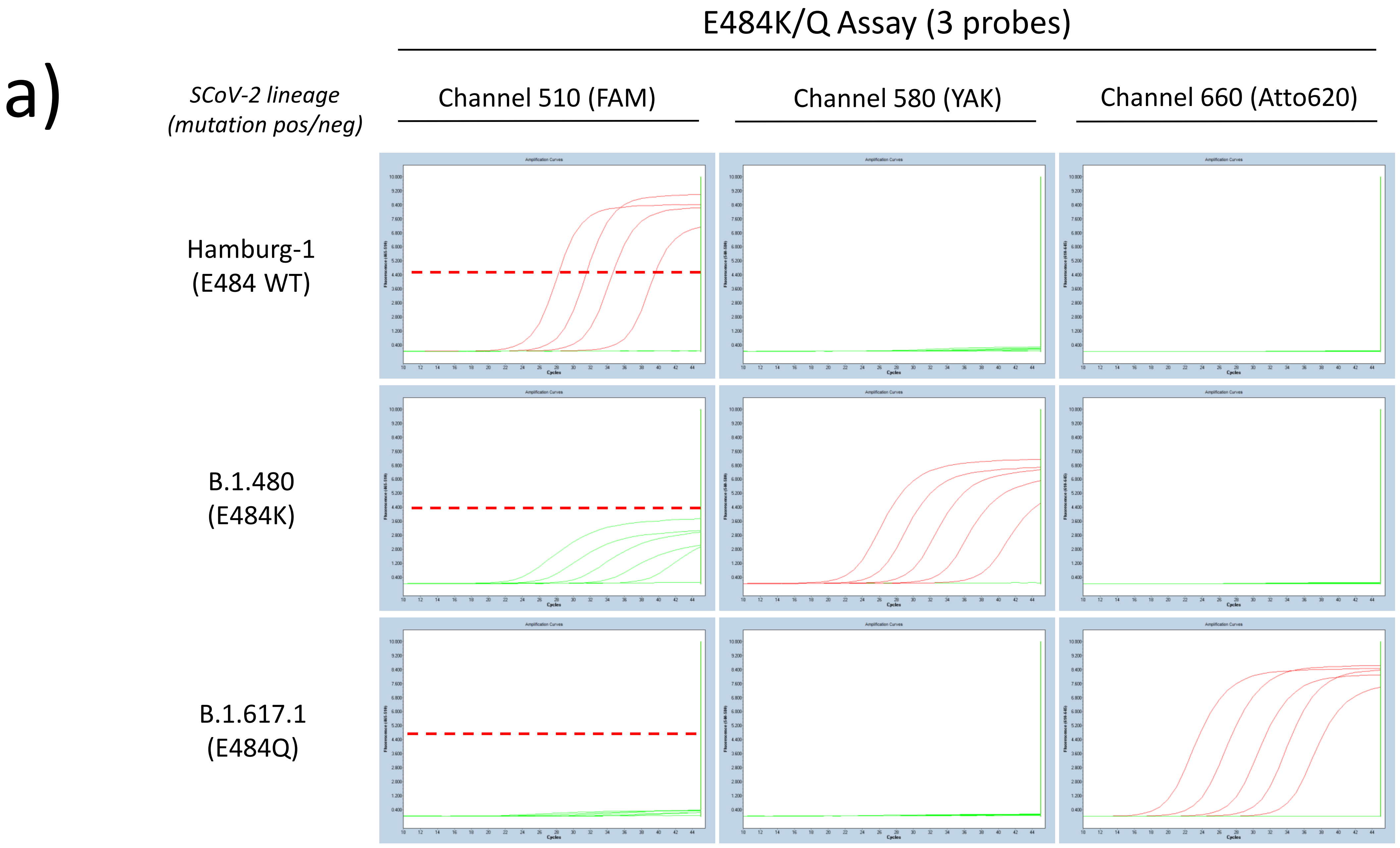

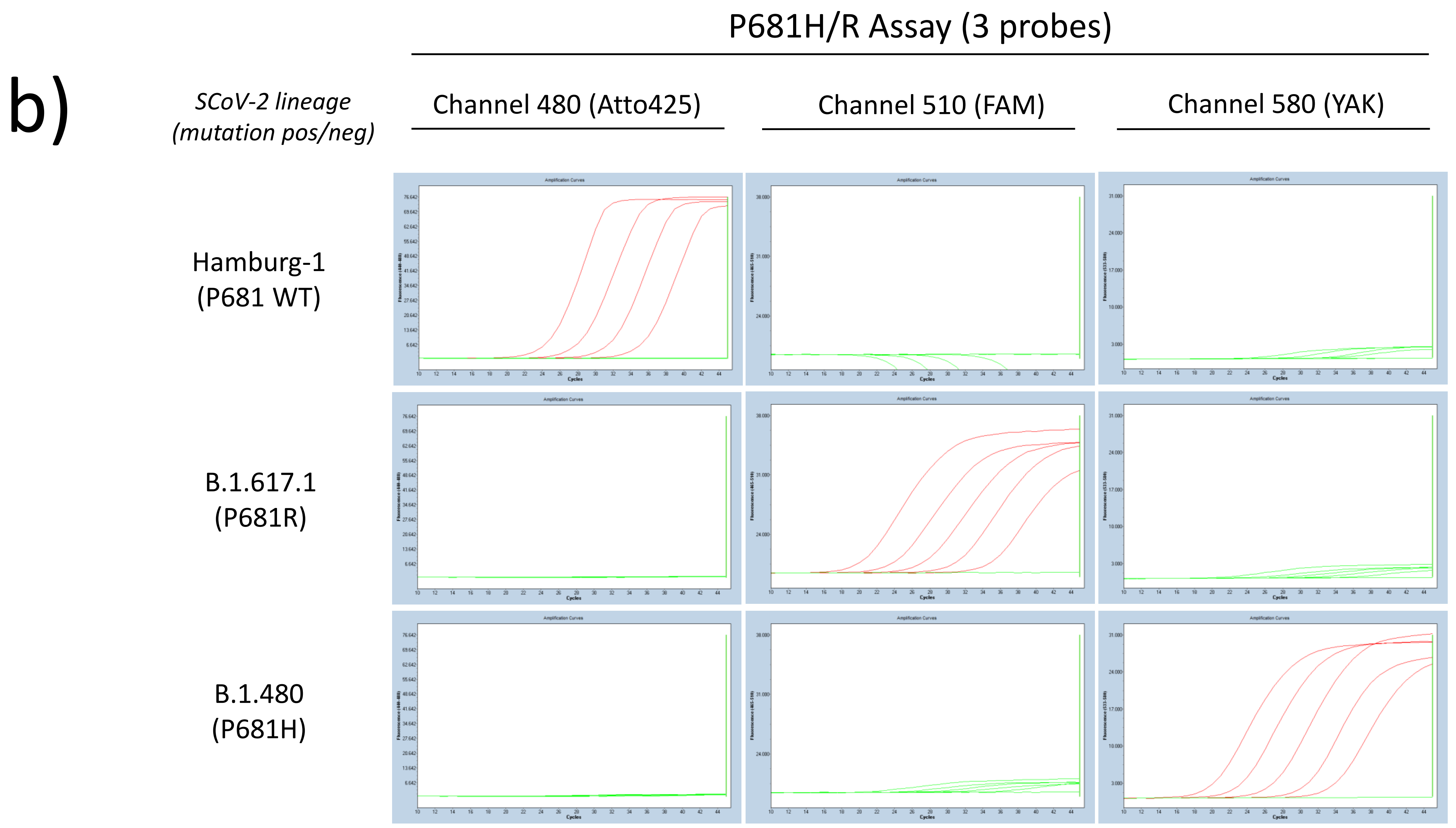

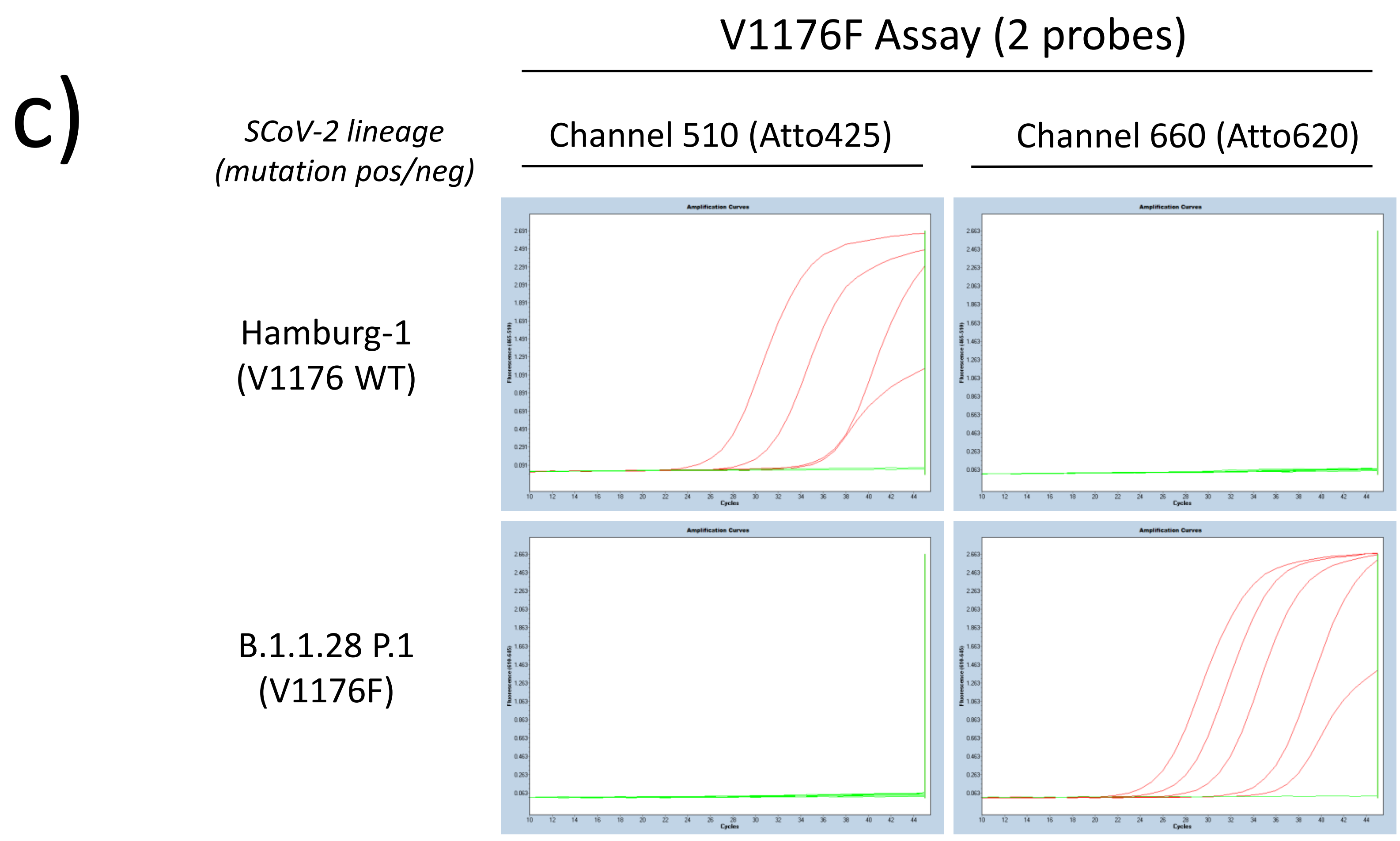

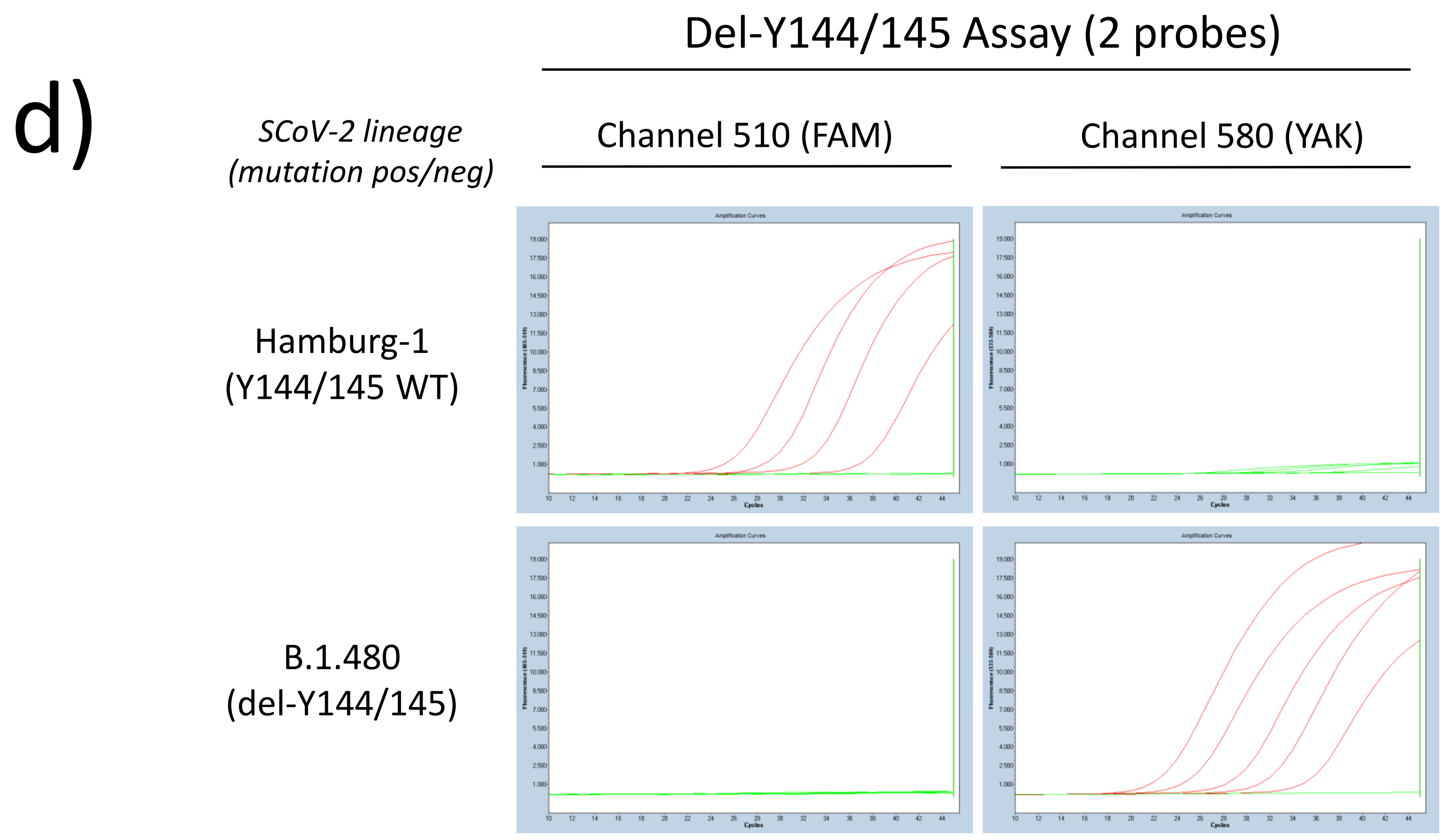

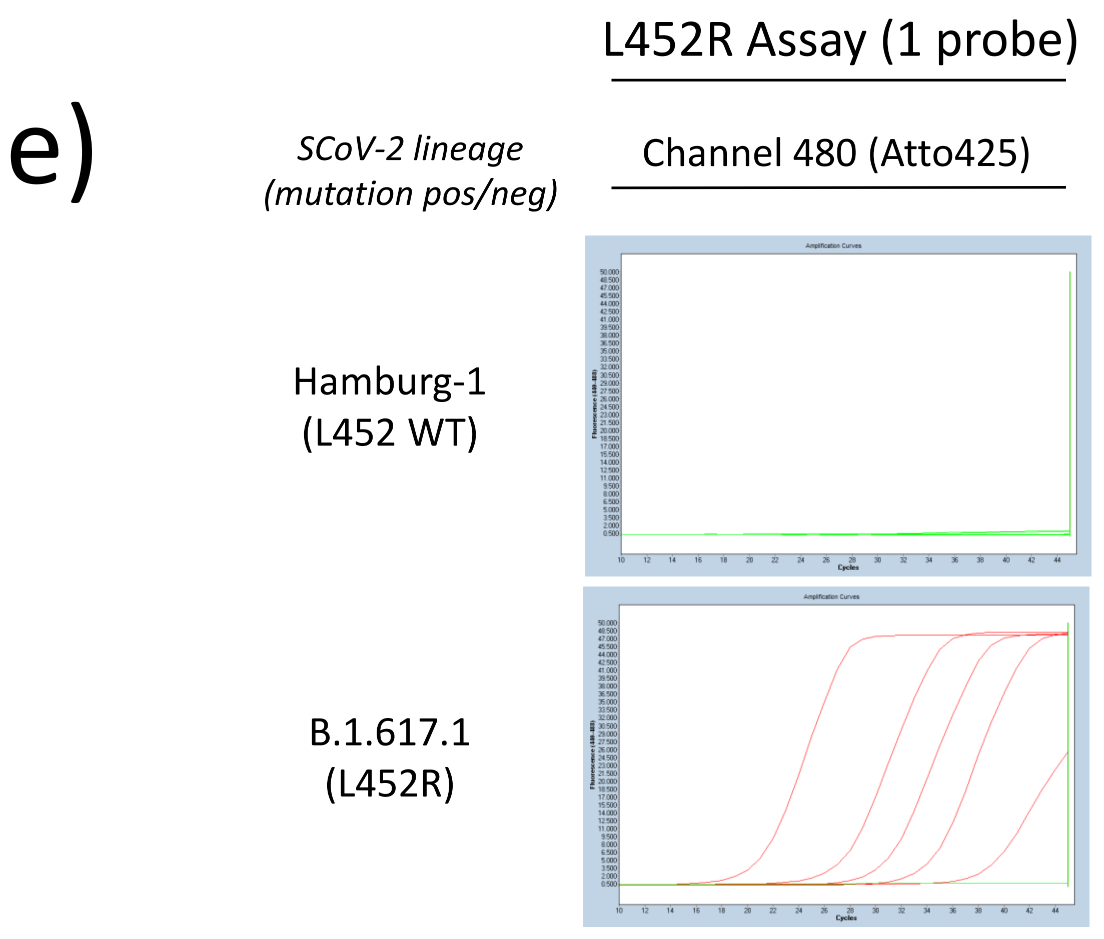

Supplementary figure S1:
Amplification curves of all SNP assays as seen in supplementary table 1. Clinical- or cell culture isolates containing respective SNPs or " wild-type" sequence were used to create 10-fold dilution series. All assays were tested with cobas omni utility channel kit mastermix and in a total of 3 repeats per dilution step. Nucleic acid extraction was carried out using a MagNA-pure96 instrument (Roche, Switzerland)
