# Supplementary material for: Rapid Automated Screening for SARS-CoV-2 B.1.617 Lineage Variants (Delta/Kappa) through a Versatile Toolset of qPCR-Based SNP Detection
Source: Diagnostics (Basel). 2021 Oct 1;11(10):1818. doi: 10.3390/diagnostics11101818 (PMC8534837; doi:10.3390/diagnostics11101818)
Supplement: Supplementary file 1 [file diagnostics-11-01818-s001.zip › diagnostics-1376430-supplementary/210922_supplement_tables.pdf]

Supplementary table S1

Target-SNP	Sequences 5' - 3'	also works with RNA process ctrl kit	Clinical validation
del-HV69/70	Fwd: TCA ACT CAG GAC TTG TTC T(OMe-U)A C<br>Rev: TGG TAG GAC AGG GTT AT(OMe-C) AAA C<br>P-HV69-70WT: TGG TCC CAG AGA CAT GTA TAG CAT<br>P-delHV69-70: TGG TCC CAG A(+G)A T(+A)G C(+A)T	Yes	[15]
N501Y	Fwd: CCG GTA GCA CAC CTT G(OMe-U)A AT<br>Rev: AGT TGC TGG TGC ATG TA(OMe-G) AA<br>P-N501WT: CC+A (+A)CC CAC (+T)(+A)(+A) TGG T(+G)<br>P-N501Y: CC(+A) (+A)CC CAC (+T)(+T)(+A) TGG T(+G)	Yes	[15]
E484K/Q	Fwd: CTA TCA GGC CGG TAG (OMe-C)A<br>Rev: GTT GGA AAC CAT ATG ATT GTA AA(OMe-G) G<br>Rev-2: AGT TGC TGG TGC ATG TA(OMe-G) AA<br>P-E484WT: AT(+G) GTG T(+T)(+G) (+A)AG (+G)TT<br>P-E484K: AT(+G) GTG T(+T)(+A) (+A)AG (+G)TT<br>P-E484Q: AT(+G) GTG T(+T)(+C) (+A)AG (+G)TT	Yes	This study
P681H/R	Fwd: TGC AGG TAT ATG CGC TAG T(OMe-U)A<br>Rev: GTG ACA TAG TGT AGG CAA TGA (OMe-U)G<br>P-P681WT: TAA (+T)TC T(+C)(+C) (+T)CG GCG<br>P-P681H: TAA (+T)TC T(+C)(+A) (+T)CG G(+C)G<br>P-P681R: A(+T)T CT(+C) (+G)(+T)C GGC G	Yes	This study
L452R	Fwd: GAT T(+C)T AAG GTT GGT GG(2OMe-U) AAT<br>Rev: TTT CAG TTG AAA TAT CT(+C) TC(2OMe-U) C<br>Rev-2: AGT TGC TGG TGC ATG TA(OMe-G) AA<br>P-L452WT: T(+T)A C(+C)(+T) (+G)TA TAG ATT (+G)TT TA(+G)<br>P-L452R: T(+T)A C(+C)(+G) (+G)TA TAG ATT (+G)TT TA(+G)	(Yes)*	This study
V1167F	Fwd: TAG GTG ACA TCT CTG GCA T(OMe-U)A<br>Rev: CAA GTT CTT GGA GAT CGA T(OMe-G)A G<br>P-V1176WT: TG(+C) TT(+C) (+A)(+G)(+T) TGT AAA CA(+T)<br>P-V1176F: TG(+C) TT(+C) (+A)(+T)(+T) TGT AAA (+C)A(+T)	Yes	-
del-Y144/145	Fwd: GAC CCA GTC CCT ACT TAT (2OMe-U)G<br>Rev: ACT CTG AAC TCA CTT TC(2OMe-C) AT<br>P-144/145WT: TTT GGG TGT (+T)(+T)(+A) (+T)(+T)(+A) CCA C<br>P-delY144/145: T(+T)T GGG TGT (+T)(+T)(+A) CCA (+C)	Yes	-

Various assays (LDTs) for the cobas omni utility channel kit as presented in this and previous studies. Ome-N, 2‘O-methyl-RNA base. (+N), Locked nucleic acid base. * The L452R assay also works with RNA process control kit but CTs were severely delayed.

Supplementary table S2

cobas omni utility channel kit

Step	Temp. [°C]	Duration [mm:ss]
UNG	50	02:00
	95	00:05
Pre-PCR	55	02:00
	60	06:00
	65	04:00
PCR1 *(5 cycles)*	95	00:05
	55	00:30
PCR2 *(45 cycles)*	91	00:05
	58	00:25
Cooling	60	01:00

Roche one-step RT-PCR Master

Step	Temp. [°C]	Duration [mm:ss]
RT	50	20:00
Denature	95	05:00
Amplification *(45 cycles)*	95	00:15
	60	00:30
Cooling	40	00:30

LightCycler480 run protocols used for proof-of-concept experiments, see supp. figure 1. Configuration was done within the LightCycler480 software (Roche diagnostics, Switzerland)

Supplementary table S3

External Quality Control Panel<br>Species	Number tested	Target: L452R	Target: P681R	Target: E484K	Target: E484Q
Influenza A H1N1 pdm09 (A/California/60/2008)	1	Negative	Negative	Negative	Negative
Influenza B Victoria (B/Brisbane/60/2008)	1	Negative	Negative	Negative	Negative
RSV-A	1	Negative	Negative	Negative	Negative
RSV-B	1	Negative	Negative	Negative	Negative
hCoV - OC43	1	Negative	Negative	Negative	Negative
MERS-CoV	1	Negative	Negative	Negative	Negative
Parainfluenzavirus-2	1	Negative	Negative	Negative	Negative
Parainfluenzavirus-3	1	Negative	Negative	Negative	Negative
Human Metapneumovirus (Subtype A)	1	Negative	Negative	Negative	Negative
Rhinovirus	1	Negative	Negative	Negative	Negative
SARS-CoV (Frankfurt-1, cell culture supernatant)	1	Negative	Negative	Negative	Negative
**Clinical samples**<br>Species		Target: L452R	Target: P681R	Target: E484K	Target: E484Q
hCoV - HKU1	1	Negative	Negative	Negative	Negative
hCoV - NL63	1	Negative	Negative	Negative	Negative
Adenovirus	2	Negative	Negative	Negative	Negative
Boca-virus	2	Negative	Negative	Negative	Negative
Parainfluenzavirus 3	1	Negative	Negative	Negative	Negative
Human Metapneumovirus	2	Negative	Negative	Negative	Negative
Rhino-/Enterovirus	3	Negative	Negative	Negative	Negative
Mycoplasma pneumoniae	1	Negative	Negative	Negative	Negative
Pneumocystis jirovecii	1	Negative	Negative	Negative	Negative

Exclusivity panel tested with the SCOV2-617VOC-UCT. No false positives occured.
